# Building capacity in quantitative research and data storytelling to enhance knowledge translation: a training curriculum for peer researchers

**DOI:** 10.1186/s40900-022-00390-6

**Published:** 2022-12-06

**Authors:** Jason M. Lo Hog Tian, James R. Watson, Megan Deyman, Billy Tran, Paul Kerber, Kajiko Nanami, Deborah Norris, Kim Samson, Lynne Cioppa, Michael Murphy, A. Mcgee, Monisola Ajiboye, Lori A. Chambers, Catherine Worthington, Sean B. Rourke

**Affiliations:** 1 MAP Centre for Urban Health Solutions, Unity Health Toronto, Toronto, Canada; 2 Institute of Medical Science, University of Toronto, Toronto, Canada; 3 Factor Inwentash Faculty of Social Work, University of Toronto, Toronto, Canada; 4 School of Public Health and Social Policy, University of Victoria, Victoria, Canada; 5 Department of Psychiatry, University of Toronto, Toronto, Canada

**Keywords:** HIV, Community-based research, Community-based participatory research, Patient engagement

## Abstract

**Background:**

Many community-based HIV research studies incorporate principles of greater involvement and meaningful engagement of people living with HIV (GIPA/MEPA) by training people with HIV as peer researchers. Unfortunately, there are still some aspects of research (e.g., quantitative data analysis and interpretation) where many projects fall short in realizing GIPA/MEPA principles. To address these gaps, we developed an eight-week training course that aimed to build the capacity of peer researchers around the understanding and interpretation of quantitative data and incorporating lived experience to increase the impact of the knowledge transfer and exchange phase of a study.

**Methods:**

Peer researchers (n = 8) participated from British Columbia, Alberta, and Ontario and lessons learned from the training were implemented throughout the dissemination of research findings from the People Living with HIV Stigma Index study. This paper presents the curriculum and main training components, course evaluation results, and challenges and lessons learned. The manuscript was created in collaboration with and includes the perspectives of both the peer researchers involved in the training, as well the course facilitators.

**Results:**

Throughout the course, peer researchers’ self-assessed knowledge and understanding of quantitative research and data storytelling improved and, through interactive activities and practice, they gained the confidence to deliver a full research presentation. This improved their understanding of research findings, which was beneficial for discussing results with community partners and study participants. The peer researchers also agreed that learning about integrating lived experience with quantitative data has helped them to make research findings more relatable and convey key messages in a more meaningful way.

**Conclusions:**

Our training curriculum provides a template for research teams to build capacity in areas of research where peer researchers and community members are less often engaged. In doing so, we continue to uphold the principles of GIPA/MEPA and enhance the translation of research knowledge in communities most greatly affected.

**Supplementary Information:**

The online version contains supplementary material available at 10.1186/s40900-022-00390-6.

## Plain English Summary

Engaging patient groups or community members is commonplace in HIV research where people living with HIV are trained as peer researchers. There are still however some gaps where community members are less engaged, especially in quantitative data analysis. This presents a barrier preventing them from being meaningfully engaged in research about them. To build capacity in these areas, we designed an eight-week online course that taught peer researchers about quantitative data analysis and interpretation with a focus on concepts that would be important for talking about key messages from research findings. This was used to enhance the knowledge translation and dissemination initiatives for the People Living with HIV Stigma Index study—a survey tool containing quantitative measures examining stigma and related health factors. Peer researchers agreed that their knowledge and understanding of the key quantitative data concepts improved significantly throughout the course. This increased understanding helped them discuss quantitative data with community members and study participants, which was important to ensure that research findings reach the affected communities. Peer researchers also agreed that incorporating their new data analysis knowledge with existing lived experience helped them to make findings more relatable and understandable which is critical for translating knowledge to other researchers and policy makers. Overall, our training curriculum gave peer researchers the confidence to talk about quantitative data and improve their capacity to disseminate research. This work also provides guidelines for training peer researchers and ensuring that they are meaningfully engaged in research studies they are a part of.

**Supplementary Information:**

The online version contains supplementary material available at 10.1186/s40900-022-00390-6.

## Background

### The role of community in community-based research

Community-based research (CBR) is a process that brings researchers and community members together to respond to issues facing communities through collaborative and inclusive research approaches [1, 2]. CBR focuses on building partnerships, shared decision making, reciprocal learning and mutual ownership of research findings among community members and academic researchers [3, 4], leading to increased recruitment and decreased attrition rates, as well as improved relationships with communities [5–7]. CBR also emphasizes the rapid translation of research findings to actionable solutions, such as increased understanding of an issue or phenomenon, the development and/or implementation of an intervention strategy, and policy and programmatic changes that benefit the community [3].

### Meaningfully engaging communities in HIV research

One field that has been particularly effective in implementing CBR principles is HIV research, which is guided by the principles of GIPA/MEPA (the greater involvement and meaningful engagement of people living with HIV/AIDS) to define the roles and responsibilities of healthcare providers, researchers, the general public, and people living with HIV (often called “peer researchers” and/or “peer research associates”) in HIV-related responses [8, 9]. While there has been work done to develop frameworks to guide these CBR approaches and evaluation tools [10–14], there is a lack of consistency across research studies, making it difficult to compare their impact in the field. There are also inconsistencies around the degree of engagement in various stages of research processes, especially in the data analysis and knowledge translation phases in HIV research [3, 15–17]. With most peer engagement in analysis being with qualitative studies, there is a need for increased efforts around building capacity in quantitative research [5, 15]. In studies driven by quantitative data, engaging peers in the data analysis can allow the team to combine their lived experience perspective with the perspectives of other researchers to analyze data and develop findings that will be most applicable and understandable to communities that the study is trying to reach [4]. This can also deepen peer researchers’ understanding of the research findings which may allow them to better lead knowledge translation and dissemination phases of a study.

### The new need for online learning in the context of COVID-19

COVID-19 lockdowns around the world have reduced the number of research studies where community members are involved, despite the capacity for them to make meaningful contributions to policy making and building public trust [18]. With pandemic restrictions preventing the implementation of capacity building activities to train peer researchers that would usually take place in-person, there is an urgent need to develop training resources that can be delivered online. It is important to continue to train peer researchers despite barriers preventing in-person gatherings to ensure they do not get left behind and that they can remain meaningfully involved, especially as many other research activities shift to an online environment. Research suggests that appropriately designed online education can produce as good or better results than face-to-face learning, and there is a desire for some online education to continue post pandemic [19–22]. More research is needed to understand how to effectively adapt training and education in the context of COVID-19.

### Objectives of the manuscript

To increase the engagement of peer researchers and to build capacity in areas where they are less engaged, we developed an eight-week online course that supported peer researchers to improve their understanding of data analysis, interpretation, knowledge translation, and presentation skills. The need for this course was born out of the peer researchers’ desire to be more equitably involved in a quantitative study they were a part of, but feeling they lacked the knowledge and confidence to talk about quantitative data. We aimed to build a foundational knowledge base that could be combined with the peer researchers’ lived experience to enhance the dissemination of research findings from the People Living with HIV Stigma Index study [23]. The goals of this manuscript are to: (1) describe our training process and the key components of the training; (2) share evaluative outcomes and determine the impact that the training had on the peer researchers; and (3) discuss the challenges and lessons learned regarding meaningful engagement in CBR. This manuscript was created in collaboration with the peer researchers involved in the training, as well as the four members of the course facilitation team and incorporates the thoughts and opinions of the group.

## Methods

### Training participants

Eight peer researchers (two from British Columbia, two from Alberta and four from Ontario) participated in the eight-week online synchronous (delivered in real time) course from February to March 2021. The peer researchers were employed by various regional study teams conducting the People Living with HIV Stigma Index study [23] and were chosen to participate in this training based on their interest and availability to commit to the training program. Given the course was delivered completely online, all participants were required to have a computer with internet connection. Training participants had varying years and levels of experience in working with data and figures in CBR studies. The number of years engaged in CBR activities ranged from 1 to 15 years and those with prior experience in research were mostly involved in data collection and engaging community members through presentations that incorporated their lived experience. There were four training facilitators including two graduate students with research and data analysis expertise, two research coordinators engaged in knowledge translation and HIV stigma-related research activities, one of whom identifies as a person living with HIV.

### Training format

Peer researcher training in CBR is commonly held face-to-face, however; due to COVID-19 pandemic restrictions, this course was offered completely online. The course curriculum was based on a cycle of experiential learning [24–26] where participants were taught new concepts through online synchronous lessons and guest lectures, reflected on these concepts with a lens of lived experience, integrated these concepts into their existing knowledge and lived experience through group discussions, and applied these concepts in the real world through weekly homework assignments and practice presentations. The course was also designed through a lens of adult learning theory where participants set their own goals that were practical and applicable to their ongoing work, built on their existing experience, and learned by creating new experiences [27, 28].

The synchronous lessons took place twice a week, each for 2.5 h, over the course of the eight-week period, with homework for most lessons with a time commitment of approximately 1 h. Peer researchers were compensated for attending the training sessions and completing homework assignments at the industry rate [29]. Lessons did not cover practical aspects of conducting data analysis (e.g., using statistical software or coding), but focused on understanding the *process* of conducting data analysis. This included learning how to ask an analyzable research question, understanding how and why we clean and recode data, summarizing data in tables and basic figures such as histograms and pie charts, and examining relationships between variables using measures of central tendency as well as simple statistical analyses such as chi-squared tests. Lessons also covered fundamental quantitative data concepts as they applied to talking about research findings including basic data terminology, types of variables, and statistical significance (see Table 1 for full curriculum). The course content was developed by the course facilitators based on the peer researchers’ areas of interest and from prior experience running smaller scale workshops with a similar target audience and content. The course materials were adapted and improved from these previous workshops based on participant feedback and tailored to the needs of the new cohort. We decided to transition these workshops into a more formal and long-form course based on peer researcher enthusiasm for the course content and the research team felt that building a foundational understanding of quantitative data was an important step to equitably involving peer researchers in a quantitative study.

**Table 1:** Training curriculum (each class runs for 2.5 h; homework approximately 1 h of work)

Phase	Class	Activity	Format
1—Training overview and stigma theory	1	Constructive feedback	Presentation and discussion
		Present overview of HIV Stigma Index in your region	Homework presentation
		**Homework:** Prepare a story based on lived experience on how social support has impacted your journey with HIV stigma	
	2	Present lived experience stories focused on social support	Homework presentation
		What is stigma? Stigma theory and types of HIV stigma	Guest lecture
		**Homework:** Review the HIV Stigma Index survey from your region	
2—Understanding quantitative data	3	Research data terminology and asking analyzable questions	Presentation and discussion
		**Homework:** Reflecting on lived experience, think of an analyzable research question about social support	
	4	Share homework research questions	Homework presentation
		The data analysis process	Presentation and discussion
		**Homework:** Develop a figure that answers your research question from the previous homework assignment (Due Class 6)	
	5	Statistical significance and understanding visual representations of data	Presentation and discussion
		**Homework:** Same as previous assignment	
	6	Present homework figures answering your research question	Homework presentation
		How data can resonate on a personal level	Guest lecture
		**Homework:** Present on a figure from your region and tell a resonating story that connects your lived experiences with the data	
3—Developing a research presentation	7	How a data figure resonates on a personal level	Homework presentation
		Telling a data driven story and integrating personal experiences into KTE	Guest lecture
		**Homework:** Combine your lived experience with data to tell a story focused on social support	
	8	Present lived experience/data presentations	Homework presentation
		Discuss final presentation topic and review draft slides	Interactive activity
		Introducing yourself and the study	Presentation and discussion
		**Homework:** Prepare to introduce yourself and the study	
	9	Practice introducing yourself and the study	Homework presentation
		How to talk about demographics and group differences	Presentation and discussion
		**Homework:** Work together by region to summarize and present your regional demographic data	
	10	Present demographic data from your region	Homework presentation
		Review main presentation slides and run through presentation	Interactive activity
		**Homework:** Prepare to present a full research presentation	
	11	Share final presentations and take questions	Homework presentation
4—Online presentation skills and KTE	12	Practice main presentation	Facilitated discussion
	13	What is KTE; social media as a communication tool	Presentation and discussion
		Explore The Positive Effect website https://www.positiveeffect.org/	Interactive activity

After course completion, the peer researchers incorporated what they had learned, using data from the People Living with HIV Stigma Index, into a 60-min research presentation (45-min presentation, 15-min question period) at the REACH Nexus National Stigma Research Committee meeting on April 22, 2021. This committee has a membership of over 100 researchers and community members and meets bimonthly to discuss HIV-related research initiatives and emerging data in Canada. The data analysis for this presentation was conducted in collaboration with the peer researchers where they came up with research questions they were interested in, data analysts helped to generate figures to answer their question, and the research team discussed together with the peer researchers about interpreting the figures and weaving in their lived experience. A similar process will be followed for any future knowledge translation and dissemination activities.

### Course evaluation

After the course concluded, an anonymous online survey was given to the peer researchers to evaluate the components of the training, reflect upon the course experience, and to determine if the course was successful at teaching the desired course material. The evaluation included 34 questions (28 7-point Likert scales and 6 open-ended) and examined the peer researchers’ overall satisfaction with the course, self-assessed knowledge and understanding of key concepts before and after the course, and suggestions for future improvement. We also held two focus groups with both the peer researchers and the course instructors to collaboratively reflect on our experience in the course and discuss key messages that the team wanted to focus on in this manuscript. Included throughout the manuscript are excerpts and reflections captured from both the peer researchers and the facilitators from the course evaluation survey and the two focus groups.

## Results

### Key training components

When developing the course curriculum, several key training components emerged. This section will elaborate on each of these components and their importance to the training success.

#### Creating a safe and engaging online space

One of the first considerations when designing the course was ensuring that peer researchers with varying backgrounds, skill levels, and experience with research felt safe in the learning environment and supported to succeed. To accomplish this, facilitators and peer researchers contributed to the development of “ground rules” that made expectations clear around creating a supportive environment that was encouraging of questions, and varying opinions and experiences (see Additional file 1: Appendix A for ground rules).

A “Slack Learning Hub” was created to extend the positive learning environment outside of the classroom. Slack is a communication platform where teams can create “channels” organized by topic, engage in discussions, and share ideas and resources (www.slack.com). Using Slack as a central space to post lecture materials and updates, ask questions (both to the group and privately using direct messaging), and have group discussions was an invaluable organizational tool for running the course. One peer researcher added: “It was useful to know that we would always be able to find something we needed in the appropriate channel, rather than having to search through our emails.”

Slack was also used to organize “office hours” where peer researchers could sign up for times to meet with the facilitators to discuss any outstanding questions from the previous session(s) and get additional support with completing the homework assignments. One peer researcher reflected on enjoying the opportunity to collaborate with facilitators one-on-one without having to worry that they were taking time away from other participants in a group setting. He commented, “This component of the training empowered me to fully understand the findings of my research question, which led to a greater confidence in presenting them to others. It also improved my relationship with the facilitators and helped create a greater sense of team support.”

#### Providing constructive feedback

Many homework assignments involved having the peer researchers develop and deliver various parts of a research presentation. To extract the most benefit from these homework presentations, the peer researchers provided and received constructive feedback on their presentations from their peers and the facilitators. It was important to create a judgment-free environment and provide a template for giving feedback during the first class. As a group, we discussed the distinction between criticism and feedback, how to take a positive and respectful approach when offering constructive feedback (the “Sandwich Method”), and how to react and respond to feedback respectfully (see Additional file 1: Appendix B for constructive feedback handout). The peer researchers improved greatly at giving and receiving constructive feedback and they agreed that it was a difficult skill to learn, yet so important. One peer researcher explained:

We are often uncomfortable with criticizing someone else, but it’s the more critical or challenging feedback that allows people to change and to realize what needs improvement. Learning how to give more critical feedback in a way that doesn’t hurt or anger people but allows them to see where they might improve was a valuable part of the training.

Giving constructive feedback also encouraged a greater level of active listening during homework presentations and hearing participants offer feedback on each other’s presentations in a group setting allowed for further opportunities to learn and integrate course material and concepts. Establishing this continuous feedback structure also encouraged further self-reflection and growth:

Every now and then someone would give me a nugget of something that really helped me think about how I could improve, which is valuable for someone who has been working in research for a long time. I found that with all the different presentations that we did throughout the training, we got a lot of honest feedback that changed my perspectives on things.

#### Building quantitative data skills

Given the time constraints of a single course, designing the curriculum for participants with varying experience in quantitative skills was challenging. Focus was put on developing skills that would be directly applicable to the peer researchers’ work with knowledge translation and engaging community members and research participants with research findings. It was important to leave time for questions and discussion while acknowledging that this course could only cover high-level concepts that gave the peer researchers the foundational knowledge required to talk about the data.

While there were inevitably gaps in their understanding, the peer researchers felt that their knowledge and understanding of quantitative data concepts had grown significantly. One peer researcher explained, “Broadening my understanding of data was so important because I realized that before the training when I attended conferences or meetings and data was being presented, to me it was just jargon because I never really understood what it was.” Another PRA added: “Now I have a more in-depth understanding of how to interpret data and figures. If I have an opportunity to be at a conference and someone is presenting data, I will now be able to understand much better than I would have before.” Many of the training participants agreed that understanding the results and having the confidence to explain them to others will be beneficial when promoting studies to community partners and when discussing study results with study participants.

#### Data storytelling

Another major aspect of the training was data storytelling, which involved the peer researchers using their lived experience to add context to the data and form a coherent narrative, allowing it to be better translated to researchers, policy makers, and community members [30–32]. Using data storytelling makes research findings more compelling, facilitates understanding, leaves a bigger impression, and most importantly, moves people to action [30]. This is an important skill for peer researchers since they play a critical role in the HIV response, especially through storytelling, to encourage governments, policy makers, and other stakeholders to act.

Many peer researchers felt the training improved their ability to share information, especially data:

Telling my story, or the story of others living with HIV whom I interviewed, allowed me to make the data relatable using terms that everyone could understand and bring it to life. I had already been incorporating storytelling into my research presentations, but the training really helped me hone that and think about how lived experience could convey our message in a more meaningful way.

Another peer researcher explained:

It was critical to talk about stories and things that we’ve learned from participants because the data that we’re presenting are their stories. It’s important that the audience knows this isn’t just a graph—these are stories about people’s lives and their stories need to be heard and respected.

#### Homework and self-learning

The course instructors relied heavily on homework to reinforce the lessons learned and to practice working with new knowledge and concepts. Most of the homework assignments involved the peer researchers working with a concept taught in the previous lesson and applying it to research findings or their data storytelling approach. Then at the following lesson, the peer researchers would give a short presentation demonstrating their completed homework assignment. While the time commitment was significant, the peer researchers felt that the homework helped to increase their knowledge and reinforce their understanding of the course materials:

The homework helped to motivate me to get prepared and learn the course materials. It was also a good opportunity to make connections outside of the training. I was able to work with a partner to pull something together and being able to talk to someone else who was also going through the training about how to present the data was helpful.

In cases where the homework assignments were challenging and/or the homework expectations were unclear, the peer researchers were encouraged to attend weekly scheduled office hours to seek additional one-on-one support from the training facilitators:

There were times when the turnaround for the homework was difficult, especially if we were working on data slides, but the level of homework support was great. The practice and help received during office hours helped me to learn how to make the presentation more understandable and incorporate storytelling to make it more interesting to people.

Overall, using homework assignments that encouraged the application of concepts learned in training sessions created the opportunity for self-learning and the greater integration of knowledge, while providing the additional support necessary to respond to the challenges with completing the homework in a timely manner.

### Training evaluation

**Fig. 1:**
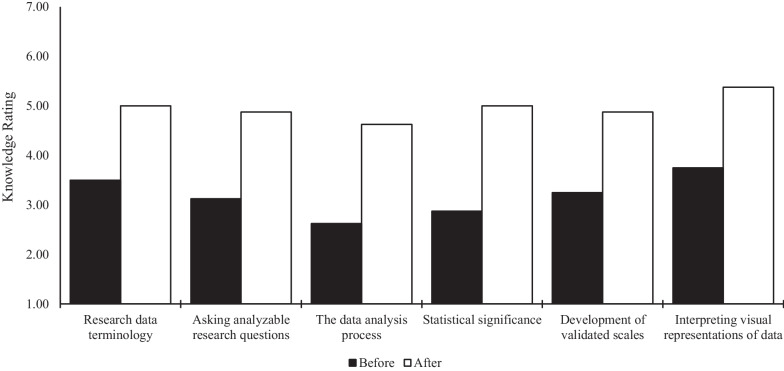
Knowledge ratings of quantitative data concepts before and after the training (n = 8). Wilcoxon signed-rank test conducted to compare pre/post test scores; all categories were significantly different (*p* < 0.05)

Peer researchers (n = 8) were satisfied with the course structure, content, and the facilitation of the curriculum. They were also satisfied with the support available, the length of the training, and the impact the training had on their work in CBR. Figure 1 shows the peer researchers’ self-assessed knowledge and understanding of key training components before and after the training. Overall, the peer researchers’ self-assessments improved on all items assessed by a consistent margin of approximately one rating point. Wilcoxon ranked-sign tests conducted to compare rating scores before and after the training showed that these improvements were statistically significant for all training components (*p* < 0.05). These findings suggest the training is beneficial to peer researchers’ comfort, while highlighting that there is still room for increasing knowledge and understanding in these areas, possibly with subsequent training opportunities. The results from the qualitative questions were used to inform the challenges section below.

## Discussion

For peer researchers to be fully engaged in quantitative research, it is important that they understand and are comfortable with quantitative data. We designed an eight-week online course to teach peer researchers data analysis and knowledge translation concepts to improve their engagement in a research study. Knowledge and understanding of key concepts increased significantly and participants felt that the training was effective at helping them integrate lived experience into research findings and improved their ability to make change. Peer researchers are often left out of the data analysis and interpretation stages of a research study despite their eagerness to be involved and the impact that could have on the dissemination of research findings [5, 6, 33]. This study aims to fill this gap by providing insight into the key components and lessons learned from developing and implementing a course to build the capacity of peer researchers in these areas that other research teams can use as guidance in their own studies. This study also provides insights into the intricacies of running an online course for peer researchers while demonstrating that they can still be successfully trained and engaged in research despite COVID-19-related disruptions [18, 21].

The study team was impressed with the peer researchers’ ability to apply the lessons learned to enhance knowledge translation activities by incorporating their lived experience to tell a story about the data. While the team had many years of CBR experience, the results of the training further reaffirmed the importance of engaging peers in research for the team. It is important to note that this course and its focus on dissemination and end of study activities is not meant to suggest we replace or shift the focus of peer researchers away from other study activities. Meaningful engagement of peer researchers is key throughout the entire research process and capacity building initiatives must be tailored to the needs and expertise of each study team. While we believe the training was an overall success, there were still some challenges that may be important to consider when planning future training and/or capacity-building opportunities. The remainder of this section will outline these challenges and some possible options for ameliorating them in the future.

### Time and flexibility

Peer researchers often have other employment and/or responsibilities in addition to their work in research, so it is important to pick a time for synchronous classes that works for everyone with room for flexibility (e.g., recorded online sessions that could be viewed later). Some peer researchers also felt pressured by the tight timelines for the homework assignments, so increasing timelines for homework completion and/or reducing the number of assignments may be beneficial for both the enjoyment of the course and for the better integration of course material. Extending the session duration by half an hour (for a total of 3 h) may address some of the issues with time constraints by allowing for more time for check-ins, breaks, discussions. and constructive feedback. More blended learning such as pre-recorded lectures and interactive, practical exercises that participants can complete on their own time may also provide more opportunities for learning while taking into consideration individual time commitments. There was also a significant time commitment for the course facilitators from developing course content together with the team, running lectures, and meaningfully supporting and collaborating with peer researchers. However, this time investment may reduce as a research team gets more experience in CBR, learns how to work with peer researchers, and can use and adapt existing capacity building tools for future work.

### Transitioning to an online environment

With COVID-19 pandemic restrictions preventing in-person training opportunities, we had to design a course that could be delivered completely online. While there were some benefits to this, such as bringing together people from across the country and more scheduling flexibility for both peer researchers and facilitators, there were also some challenges with this transition. Some peer researchers were less familiar with the tools used to deliver the online course (e.g., Zoom, Slack, Microsoft PowerPoint, Google Docs) and extra time was spent in class and during office hours teaching them how to use the tools. A short pre-course lesson or external prerequisite educational videos could be a useful strategy to get participants up to speed with the software tools required for the course. When appropriate to do so, an in-person meeting with all participants may be beneficial to further strengthen relationships and practice in-person presentation skills. As COVID-19 restrictions begin lifting, further work must be done to evaluate the potential of a hybrid model (a mix of in-person and online education) to strike a balance between increasing accessibility while still providing quality education. Additionally, while all participants in this training had access to a computer with internet service, this may not be possible for certain individuals or population groups, limiting their ability to be engaged in such courses in the future. It will be important to develop trainings tailored to the needs of such groups, possibly incorporating some aspect of in-person learning within communities, especially as COVID-19 restrictions lift.

### Support and guidance for reflecting on lived experiences

There were occasions where peer researchers shared lived experiences that were sometimes emotional or traumatic to talk about. The facilitators made sure to check-in with the peer researchers periodically about their emotional health and the peer researchers also found support from their fellow classmates who were navigating a similar process. This continued support is important to allow for more open and genuine storytelling. Formal, regular check-ins may also be helpful to provide a dedicated time to offer support around sharing lived experiences. Some CBR studies hire an external counsellor or social worker to provide support to peer researchers or the study team if needed which could be another useful resource for a peer researcher training [34]. The need for check-ins in an online space becomes more crucial when you are unable to provide physical connections (due to distance or COVID-19 restrictions).

### Planning next steps in advance

The peer researchers engaged in this training were ambitious and passionate about their work, and often expressed a strong desire to dive deeper into working with raw data, conducting data analysis, and constructing research figures and tables. Since a comprehensive education on these topics was outside the scope of this course, managing expectations and reframing the purpose of the course was important throughout the training. The peer researchers were also eager to put the lessons learned to immediate use, and while they did give a formal research presentation shortly after the training, we did not have a formal schedule of subsequent knowledge mobilization activities for them to engage in. In the future, it may be beneficial to have a concrete plan in place from the beginning for transitioning the lessons learned into a set number of presentations and/or other knowledge translation activities over the weeks and months following the completion of the course. It is also important to note that this course was designed and implemented after the study had commenced, which allowed us to use real data that the peer researchers collected as teaching tools. Depending on the goals of the training, it may be important for future studies to initiate certain capacity building activities as early as possible in the research process to give as much time as possible to plan for next steps and so lessons learned can get put into action in a meaningful way.

## Conclusion

Meaningfully engaging people living with HIV in all aspects of research will require building capacity in quantitative data and statistical analysis. Our training program has improved peer researchers’ knowledge and understanding in these areas, reduced the fear often associated with contextualizing data, and built their confidence in incorporating quantitative findings in research presentations and other knowledge translation activities. The key training components and lessons learned provide a template for research teams across many disciplines to utilize, adapt and deliver to build capacity in areas of research where peer researchers and community members are less often engaged. In doing so, we will continue to uphold the principles of GIPA/MEPA in CBR and enhance the translation and useability of research knowledge in communities most greatly affected.

## Supplementary Information

**Additional file 1**: Appendix A—Ground rules; Appendix B—Constructive feedback handout

## Data Availability

The datasets used and/or analysed during the current study are available from the corresponding author on reasonable request.

## Abbreviations

HIV: Human immunodeficiency virus
CBR: Community-based research
GIPA/MEPA: Greater involvement and meaningful engagement of people living with HIV
