# Supplementary material for: Building capacity in quantitative research and data storytelling to enhance knowledge translation: a training curriculum for peer researchers
Source: Res Involv Engagem. 2022 Dec 6;8:69. doi: 10.1186/s40900-022-00390-6 (PMC9724271; doi:10.1186/s40900-022-00390-6)
Supplement: Supplementary file 1 — Additional file 1: Appendix A—Ground rules; Appendix B—Constructive feedback handout [file 40900_2022_390_MOESM1_ESM.docx]

Appendix A – Ground rules developed collaboratively on Day 1 of the course

**PRA KTE Training: Phase 1 February 10, 2021**

Ground Rules

- **Keep an open mind**
- **Challenge the idea not the person**
- **Confidentiality**
- **Respect others and their opinions**
- **Be supportive and encourage growth and learning**
- **Realize that everyone works differently, even if it is towards the same goal**
- **Do not interrupt when others are speaking**
- **Keep to the time/be punctual/be prepared**
- **Stay mentally and physically present**
- **Give everyone a chance to speak/share the air**
- **Do the work**

Appendix B – Constructive feedback handout

**PRA KTE Training: Phase 1 February 10, 2021**

**Providing & Receiving Constructive Feedback**

### “**Constructive feedback** is providing useful comments and suggestions that contribute to a positive outcome, a better process or improved behaviours. It provides encouragement, support, corrective measures and direction to the person receiving it” (University of Tasmania, n.d.).

**Feedback is not criticism**

| **Criticism** | **Feedback** |
| --- | --- |
| Has negative effects on the receiver | Positive (constructive and planned) |
| Value- or emotionally-based | Descriptive, observational |
| Focuses on the person | Focuses on the presentation/product |
| Judgmental | Supportive |
| Problem-focused | Learning-focused and solution-focused |
| Focused on the past or present | Future oriented |

*Adapted from Department for Health and Wellbeing, Government of South Australia (2018).

# The four-part formula to giving great feedback:

## The Micro-yes

1. **Your data point**
2. **Your impact statement**
3. **Ending with a question**

**Providing balanced feedback: The sandwich method**

| **Commend** | - *I appreciate the time you spent on ...* - *In general I am really happy with your performance. I have been impressed with the way you have ...* |
| --- | --- |
| **Recommend** | - *One area where I’d like to see an improvement is ...* - *Perhaps next time around you could also ...* - *It’s not yet meeting my expectations. What I’d like to see is ...* - *One way of developing your skills even further would be to ...* |
| **Commend** | - *I feel confident that it will go better next time.* - *I appreciate your willingness to take on board feedback.* |

*(Adapted from ACT Government, n.d., p. 12)

# Receiving feedback

| **React** | - Need to be aware of and manage our emotional reaction to what we are hearing. This includes use of body language and facial expressions/ - Practice the skills of active listening, so we are listening to the feedback and asking questions to ensure that we understand the other person’s views and expectations. - Try to suspend judgement. |
| --- | --- |

|  | - Must remember not to skip the next stage! Depending on what the feedback is, we might need to end the discussion at this point to give ourselves time to think properly about what we have heard. |
| --- | --- |
| **Reflect** | - Summarize and reflect on what you heard. - Should be honest with ourselves about our own performance and be open minded about what the other person has said. - Should allow ourselves extra time for our emotions to calm down if necessary. - Ask questions to clarify, making sure you understand the feedback - Ask for specific examples to help us to understand the feedback e.g.   - “What would you have preferred me to do”?   - “How could I do it differently next time”? |
| **Respond** | - Accept the feedback by thanking the person giving it. - If we don’t agree, we respectfully say so and support what we’re saying with facts or our alternative views. - Check with others to determine the reliability of the feedback you received. - Should focus on the future and improvement - suggest options or   solutions. We respectfully negotiate and agree to ‘next steps’ (to address issues raised in the feedback).   - Need to ensure we understand the next steps and we are committed and able to implement what have agreed to do. |

*(Adapted from ACT Government, n.d., p. 12)

# References

ACT Government. (n.d.). *ACTPS Performance Framework*. Retrieved from: [https://www.cmtedd.act.gov.au/ data/assets/pdf_file/0003/463728/art_feedback.pdf](https://www.cmtedd.act.gov.au/__data/assets/pdf_file/0003/463728/art_feedback.pdf)

Brown, B. (2020). *Daring Greatly: Engaged Feedback Checklist*. Retrieved from: [https://brenebrown.com/wp-](https://brenebrown.com/wp-content/uploads/2019/06/Engaged-Feedback-Checklist-Download-2020.pdf) [content/uploads/2019/06/Engaged-Feedback-Checklist-Download-2020.pdf](https://brenebrown.com/wp-content/uploads/2019/06/Engaged-Feedback-Checklist-Download-2020.pdf)

Department for Health and Wellbeing, Government of South Australia. (2018). *Giving and Receiving Feedback*. Retrieved from: [https://www.sahealth.sa.gov.au/wps/wcm/connect/16ef08be-5701-4d74-bb41-](https://www.sahealth.sa.gov.au/wps/wcm/connect/16ef08be-5701-4d74-bb41-3123e538116f/17039.12.7%2BFG%2BGiving%2B%26%2BReceiving%2BFeedback%2BExercise%2Bv1.0.pdf?MOD=AJPERES&amp%3BCACHEID=ROOTWORKSPACE-16ef08be-5701-4d74-bb41-3123e538116f-niRmEm4) [3123e538116f/17039.12.7+FG+Giving+%26+Receiving+Feedback+Exercise+v1.0.pdf?MOD=AJPERES&amp;CACHEI](https://www.sahealth.sa.gov.au/wps/wcm/connect/16ef08be-5701-4d74-bb41-3123e538116f/17039.12.7%2BFG%2BGiving%2B%26%2BReceiving%2BFeedback%2BExercise%2Bv1.0.pdf?MOD=AJPERES&amp%3BCACHEID=ROOTWORKSPACE-16ef08be-5701-4d74-bb41-3123e538116f-niRmEm4) [D=ROOTWORKSPACE-16ef08be-5701-4d74-bb41-3123e538116f-niRmEm4](https://www.sahealth.sa.gov.au/wps/wcm/connect/16ef08be-5701-4d74-bb41-3123e538116f/17039.12.7%2BFG%2BGiving%2B%26%2BReceiving%2BFeedback%2BExercise%2Bv1.0.pdf?MOD=AJPERES&amp%3BCACHEID=ROOTWORKSPACE-16ef08be-5701-4d74-bb41-3123e538116f-niRmEm4)

Renninger, L., (2020, January). *The secret to giving great feedback* [Video]. TED Series, The Way We Work. Retrieved from: <https://www.ted.com/talks/leeann_renninger_the_secret_to_giving_great_feedback#t-2801>

University of Tasmania. (n.d.). *Constructive feedback principles*. Retrieved from: [https://www.utas.edu.au/curriculum-and-quality/student-surveys/evaluate/constructive-feedback-](https://www.utas.edu.au/curriculum-and-quality/student-surveys/evaluate/constructive-feedback-principles#%3A~%3Atext%3DConstructive%20feedback%20is%20providing%20useful%2Cto%20the%20person%20receiving%20it) [principles#:~:text=Constructive%20feedback%20is%20providing%20useful,to%20the%20person%20receiving%20it](https://www.utas.edu.au/curriculum-and-quality/student-surveys/evaluate/constructive-feedback-principles#%3A~%3Atext%3DConstructive%20feedback%20is%20providing%20useful%2Cto%20the%20person%20receiving%20it)


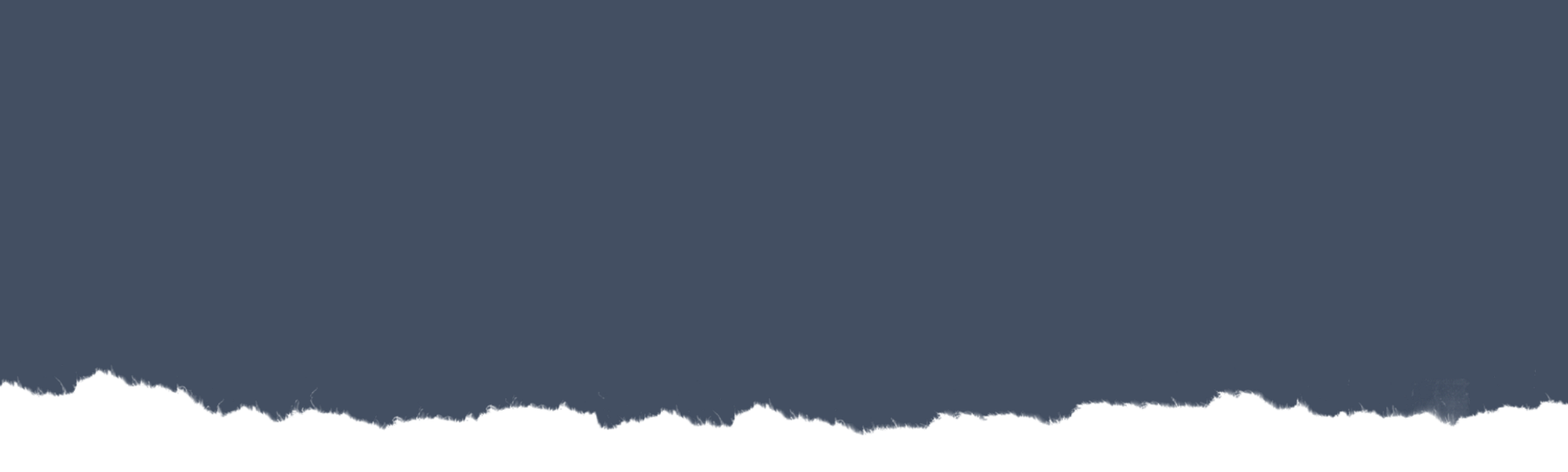


DARING GREATLY

***I know I’m ready to give feedback when:***


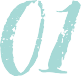
 I’m ready to sit next to you rather than across from you.


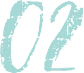
I’m willing to put the problem in front of us rather than between us (or sliding it toward you).

I’m ready to listen, ask questions, and accept that I may not fully understand the issue.

I want to acknowledge what you do well instead of picking apart your mistakes.


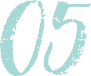
I recognize your strengths and how you can use them to address your challenges.


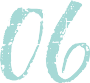
 I can hold you accountable without shaming or blaming you.

I’m willing to own my part.


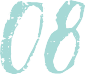
I can genuinely thank you for your efforts rather than criticize you for your failings.

I can talk about how resolving these challenges will lead to your growth and opportunity.


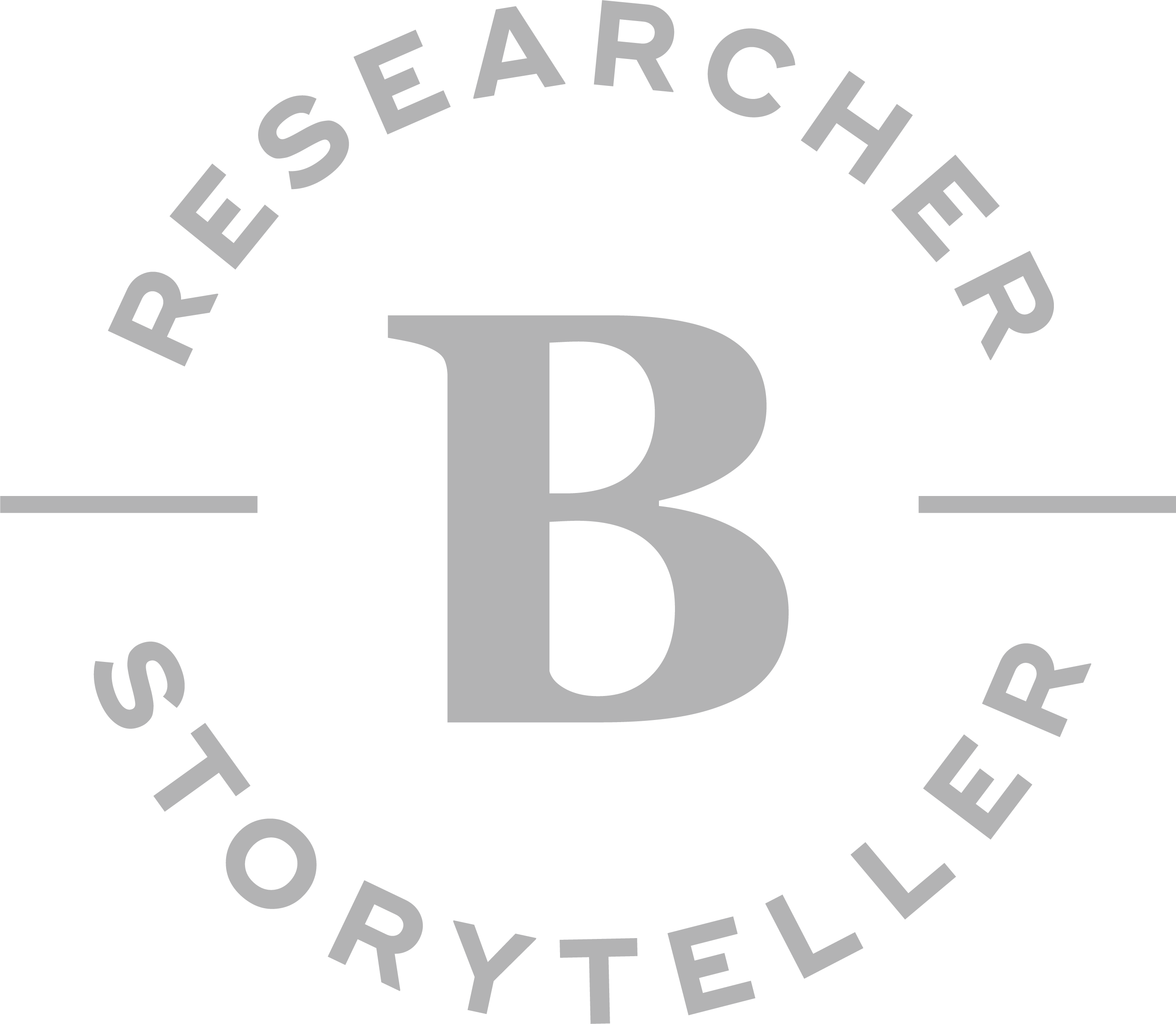

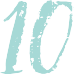
 I can model the vulnerability and openness that I expect to see from you.

from *Daring Greatly* by Brené Brown | Copyright © 2020 Brené Brown, LLC.
